# Dose-adjusted and dose/kg-adjusted concentrations of mycophenolic acid precursors reflect metabolic ratios of their metabolites in contrast with tacrolimus and cyclosporine

**DOI:** 10.1042/BSR20182031

**Published:** 2019-09-13

**Authors:** Ewa Hryniewiecka, Jolanta Żegarska, Dorota Żochowska, Emilia Samborowska, Radosław Jaźwiec, Maciej Kosieradzki, Sławomir Nazarewski, Michał Dadlez, Leszek Pączek

**Affiliations:** 1 Department of Immunology, Transplant Medicine and Internal Diseases, Medical University of Warsaw, Poland; 2 Mass Spectrometry Laboratory, Institute of Biochemistry and Biophysics, Polish Academy of Sciences, Warsaw, Poland; 3 Department of General and Transplantation Surgery, Medical University of Warsaw, Poland; 4 Department of General, Vascular and Transplant Surgery, Medical University of Warsaw, Poland; 5 Institute of Genetics and Biotechnology, Biology Department, Warsaw University, Poland; 6 Department of Bioinformatics, Institute of Biochemistry and Biophysics, Polish Academy of Sciences, Poland

**Keywords:** cyclosporine, dose-adjusted concentration, kidney transplantation, liver transplantation, metabolic ratio, tacrolimus

## Abstract

Background and purpose: Therapeutic drug monitoring is a valuable tool supporting immunosuppressive therapy. Significant variation of immunosuppressive drug (ISD) concentrations during their use at similar doses is the basis of dose-normalization strategy. The strategy of dose-adjustment is proposed to identify variability in the rate of ISD metabolism. While the parent drug-to-metabolite ratio (metabolic ratio, MR) represents the rate of formation of individual metabolites. The present study was aimed at evaluation of associations between ISDs’ metabolism rate expressed as dose-adjusted concentrations (C/D) and dose/kg-adjusted concentrations (C/D/kg) and MRs of individual metabolites of tacrolimus, cyclosporine A and MPA precursors.

Experimental approach: 506 patients have participated: 284 males (56.13%) and 222 females (43.87%); 318 after kidney (62.85%) and 188 after liver transplantation; median age was 51.34 (39.32-59.95) years and median time after transplantation 78.92 (33.87-138.4) months.

Key results: Generally, we have not observed significant relationships between dose-adjusted and dose/kg-adjusted concentrations and MRs of cyclosporine and tacrolimus. Significant correlations were found for: AM9/CsA and dMC-CsA/CsA in kidney transplant recipients and MIII/Tac, AM1/CsA and AM4N/CsA in liver transplant recipients. In contrast, MRs of mycophenolic acid (MPA) metabolites correlated significantly with MPA C/D and C/D/kg both in kidney and liver transplant recipients.

Conclusion and implications: In conclusion, easily available and easy to use in clinical practice C/D and C/D/kg ratios cannot be considered as parameters directly reflecting the rate of generation of major metabolites of cyclosporine and tacrolimus both in liver and kidney transplant recipients.

## Introduction

Therapeutic drug monitoring (TDM) is a valuable tool that supports usage of medicinal products with narrow therapeutic window and high inter- and intraindividual variability of dose/exposure relationship. Due to considerable potency of adverse and toxic effects, immunosuppressive therapy after solid organ transplantation requires application of TDM. Traditionally, immune tests are used for TDM, however, chromatography with mass spectrometry is considered the most accurate method [1].

As a natural consequence of phases I and II of drugs biodegradation, various metabolites of immunosuppressive drugs (ISD) become detectable in the blood and tissues. Some of these metabolites are toxic and may exhibit some immunosuppressive activity, but our knowledge in this field is limited. Cyclosporine A (CsA) is metabolizsed in the I phase due to CYP3A4 and CYP3A5 activity and its major metabolites are AM1, AM9 and AM4N [2]. Those three metabolites also exhibit the highest biological activity of the parent drug. Metabolism of tacrolimus (Tac) is mainly hepatic by P450 enzyme system: CYP3A4 that forms mainly 15-O-desmethyl-Tac (MIII) and smaller amounts of 13-O-desmethyl-Tac (MI) and 31-O-desmethyl-Tac (MII), and CYP3A5 that forms mainly 31-O-desmethyl-Tac and smaller amounts of 13-O-desmethyl-Tac, 15-O-desmethyl-Tac and 12-hydroxy-Tac (MIV). Mycophenolic acid (MPA) is the active metabolite of two commercially available mycophenolates (mycophenolic acid precursors (pMPA)) formed by its rapid hydrolysis. Mycophenolic acid metabolism in hepatocytes, enterocytes and renal cells leads to formation of its metabolites: 6-O-desmethyl-MPA, 7-O-MPA-glucuronide, phenyl-MPA-glucuronide (MPAG), acyl-MPA-glucuronide (AcMPAG) and 7-MPA-glucoside (GluMPA). MPAG is considered the major inactive MPA metabolite and reaches plasma concentrations approximately 20- to 100-fold higher than parent drug [3]. The awareness of the influence of genetic variability and of many other factors on the rate of ISD metabolism has become a reason to look for appropriate indicators. Significant variation of immunosuppressive drug concentrations during their use at similar doses was the basis of dose-normalization strategy. Dose-normalization was applied both to the parent drug concentrations and to metabolites’ concentrations [4–8]. Higher drug concentration/dose ratios are indicative of lower activity of complex processes leading to ISD clearance from patients’ blood, including slower metabolism, whereas higher metabolite concentration/dose ratios indicate increased rate of these processes. Calculation of metabolic ratio (MR) represents another strategy that evaluates more closely the intensity of parent drug metabolism. No uniform strategy has been developed and various authors propose different approaches, i.e. calculation of individual metabolite to parent drug concentration ratio, individual metabolite AUC to parent drug AUC ratio, and total metabolites concentration to parent drug concentration ratio [8–14].

In order to conduct a more detailed assessment of the proposed strategies, the present study was aimed at evaluation of associations between immunosuppressive agents’ clearance rate expressed as dose-adjusted concentrations (C/D) and dose/kg-adjusted concentrations (C/D/kg) and metabolic ratios of individual metabolites of tacrolimus, cyclosporine A and MPA precursors in patients after kidney transplantation (KTX) or liver transplantation (LTX).

## Experimental

The study was performed in the outpatient clinic of the Department of Immunology, Transplant Medicine, and Internal Diseases of the Medical University of Warsaw (MUW) from May 2014 to April 2016. The study protocol was approved by the MUW Ethical Committee. Before the study procedures, all patients have given their written informed consent. All procedures performed were in accordance with the ethical standards of the MUW Ethical Committee and with the 1964 Helsinki declaration and its later amendments.

The study included all patients who presented to our transplant clinic for routine planned visit, who were on stable immunosuppression therapy for at least 3 months, who have no acute health problems, and who have given their informed consent to participate in the study. From each study participant 2 ml of blood was collected during the routine outpatient visit. The blood samples were withdrawn 12 h after administration of the last ISD dose. The time interval was controlled by experienced transplant nurse staff on the basis of patients’ declarations.

Whole blood concentration of cyclosporine and its metabolites: AM1, AM9, dihydro-cyclosporine (DiH-CsA), trihydro-cyclosporine (TriH-CsA) and desmethylcarboxy-cyclosporine (dMC-CsA); tacrolimus and its metabolites: 13-O-desmethyl tacrolimus (MI), 15-O-desmethyl tacrolimus (MIII); and plasma concentrations of mycophenolic acid (MPA) and its metabolites: phenyl glucuronide (MPAG), acyl glucuronide (AcMPAG) and glucoside (GluMPA) were quantified at the Institute of Biochemistry and Biophysics using liquid chromatography-tandem mass spectrometry method (LC-MS/MS). Analyses were performed using Waters Acquity Ultra Performance Liquid Chromatograph coupled with Waters TQ-S triple-quadrupole mass spectrometer (Waters Corporation, Milford, US), as described previously [19].

Clinical and laboratory data were extracted from patients’ medical records and included in dedicated database. Data concerning patients’ body mass and ISD daily dose were extracted from patients’ medical records.

Dose-adjusted ISD concentrations and dose/kg-adjusted ISD concentrations were calculated using the following equations: ISD concentration (ng/ml)/ISD daily dose (mg/day) or (g/day) and ISD concentration (ng/ml)/ISD daily dose per body mass kilogram (mg/kg/day) or (g/kg/day), respectively. The individual ISD metabolites’ metabolic ratios (MR) were calculated using the following equation: (metabolite concentration (ng/ml)/parent drug concentration (ng/ml)) × 100%. Total concentrations and concentrations of the given immunosuppressive drug were calculated using the following equations: AM1 + AM9 + AM4N for cyclosporine A, MI + MIII for tacrolimus and GluMPA + AcMPAG + MPAG for MPA.

Data and statistical analysis: All calculations were performed using Statistica 13.1 (Dell, Texas, USA). We used the Shapiro–Wilk test to assess the type of distribution of analysed variables. Not-normally distributed variables were presented as median and interquartile range (IQR) and were analysed using Spearman’s correlation coefficient. KTX and LTX populations were analysed separately. The differences were considered statistically significant when *P* < 0.05.

## Results

A total of 506 patients have participated in the study: 284 males (56.13%) and 222 females (43.87%); 318 patients were kidney recipients (62.85%) and 188 patients were liver recipients (37.15%); steroids were taken by 369 patients (72.93%); pMPA were taken by 314 patients (62.06%); Tac was taken by 308 patients (60.87%) and CsA was taken 157 patients (31.03%). Median age with interquartile range was 51.34 (39.32–59.95) years, median time after transplantation with interquartile range was 78.92 (33.87–138.4) months.

Due to the number of medicines taken and the small group sizes for individual concomitant drugs, ISD and transplanted organs, it was not possible to analyse the relationship between individual drug groups and the parameters of ISD and their metabolites. However, analysed patients did not take many agents listed in the summary of products’ characteristics as entering the most relevant and best documented interactions, i.e. barbiturates, carbamazepine, oxcarbamazepine, phenytoin, nafcillin, intravenous sulfadimidine, probucol, orlistat, ticlopidine, sulfinpyrazone, terbinafine, bosentan, rifampicin, octreotide, metoclopramide, high doses of methylprednisolone, imatinib, colchicine, nicardipine and nefazodone. The most numerous groups of drugs taken by the patients were medicines used in the treatment of hypertension, coronary heart disease, dyslipidaemia and hyperglycaemia: loop diuretics (29.96%), thiazide diuretics (6.1%), spironolactone (5.15%), angiotensin convertase inhibitors (17.46%), angiotensin receptor blockers (6.99%), alpha-adrenergic blockers (13.95%), beta-adrenergic blockers (52.02%), calcium channel blockers (31.25%), imidazoline receptor agonists (7.9%), insulin (15.26%), oral antidiabetic drugs (5.33%) and HMG-CoA reductase inhibitors (33.82%).

**Figure 1:**
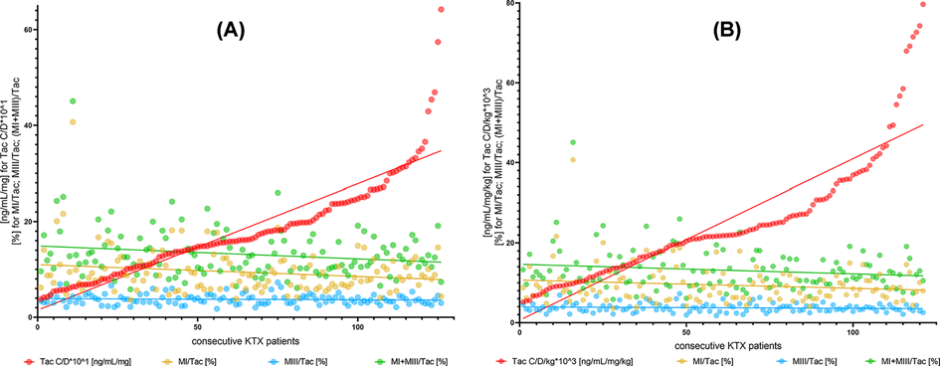
Relationship between dose-adjusted and dose/kg-adjusted tacrolimus concentrations and metabolic ratios of tacrolimus metabolites MI, MIII and MI+MIII (**A** and **B**) Lack of correlations between tacrolimus C/D and C/D/kg and metabolic ratios of MI, MIII and MI+MIII. (**C** and **D**) C/D, dose-adjusted concentration; C/D/kg, dose/kg-adjusted concentration; ISD, immunosuppressive drug; Tac, tacrolimus; MI, 13-O-desmethyl tacrolimus; MIII, 15-O-desmethyl tacrolimus; KTX, kidney transplant.

**Figure 2:**
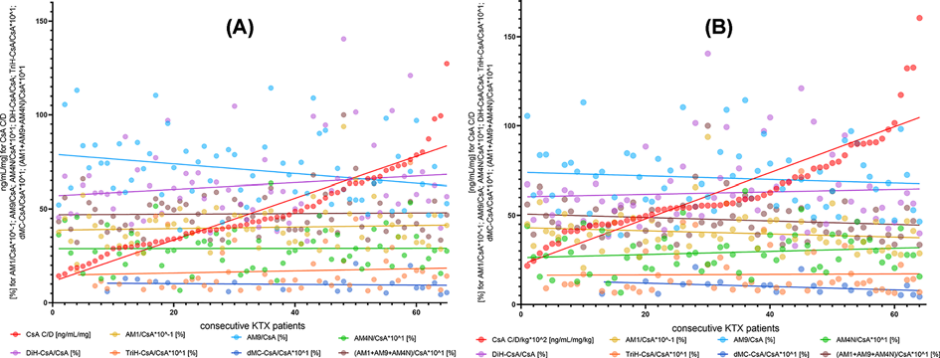
Relationship between dose-adjusted and dose/kg-adjusted cyclosporine A concentrations and metabolic ratios of cyclosporine A metabolites AM1, AM9, AM4N, dMC-CsA, dihydroxylated-CsA and trihydroxylated-CsA (**A** and **B**) Lack of correlations between cyclosporine C/D and C/D/kg and metabolic ratios of AM1, AM9, AM4N, DiH-CsA, TriH-CsA, dMC-CsA and AM1+AM9+AM4N. CsA, cyclosporine A; ISD, immunosuppressive drug; AM1, 1-hydroxy cyclosporine; AM9, 9-hydroxy cyclosporine; AM4N, 4-N-demethyl cyclosporine; dMC-CsA, demethylcarboxylated cyclosporine metabolites; DiH-CsA, dihydroxylated cyclosporine metabolites; TriH-CsA, trihydroxylated cyclosporine metabolites; KTX, kidney transplant.

**Figure 3:**
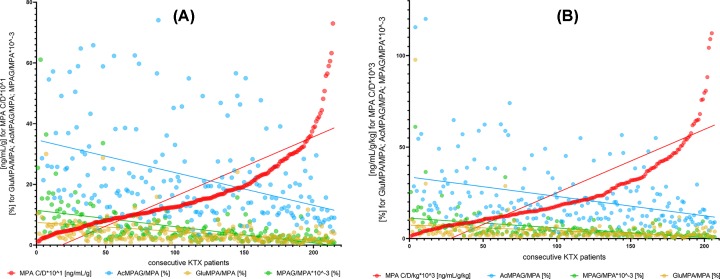
Relationship between dose-adjusted and dose/kg-adjusted MPA concentrations and metabolic ratios of MPA metabolites GluMPA, MPAG and AcMPAG (**A** and **B**) Statistically significant correlation between MPA C/D and C/D/kg and metabolic ratios of GluMPA, AcMPAG and MPAG. MPA, mycophenolic acid; ISD, immunosuppressive drug; MPA, mycophenolic acid; MPAG, phenyl glucuronide; AcMPAG, acyl glucuronide; GluMPA, glucoside; KTX, kidney transplant.

Generally, we have not observed significant relationships between dose-adjusted and dose/kg-adjusted concentrations and MRs of cyclosporine and tacrolimus (Figure 1). There were some exceptions, i.e.: AM9/CsA and dMC-CsA/CsA in kidney transplant recipients and MIII/Tac, AM1/CsA and AM4N/CsA in liver transplant recipients (Table 1 and Figure 2A,B). In contrast, different results were obtained in the case of MPA. We have observed strong relationships for both MPA C/D and C/D/kg and all MR values (GluMPA, AcMPAG, MPAG and total MPA metabolites’ concentration; Table 1 and Figure 3A,B).

**Table 1:** Spearmans’ correlations between tacrolimus, cyclosporine A and MPA precursors’ dose-adjusted and dose/kg-adjusted concentrations and metabolic ratios of cyclosporine, tacrolimus and pMPA metabolites

ISD metabolism parameter	Metabolic ratio of ISD	KTX		LTX	
		*r*	*P*	*r*	*p*
Tac C/D (ng/ml/mg)	MI/Tac	−0.1	0.26	0.12	0.22
	MIII/Tac	−0.06	0.5	−0.25	0.02
	MI+MIII/Tac	−0.13	0.16	0.05	0.64
Tac C/D/kg (ng/ml/mg/kg)	MI/Tac	−0.06	0.51	0.1	0.35
	MIII/Tac	−0.08	0.36	−0.26	0.01
	MI+MIII/Tac	−0.09	0.32	0.02	0.88
CsA C/D (ng/ml/mg)	AM1/CsA	0.01	0.95	−0.32	0.12
	AM9/CsA	−0.31	0.01	−0.38	0.07
	AM4N/CsA	−0.03	0.81	0.02	0.93
	DiH-CsA/CsA	0.09	0.48	−0.18	0.39
	TriH-CsA/CsA	0.1	0.47	−0.37	0.15
	dMC-CsA/CsA	−0.3	0.19	−0.02	0.96
	AM1+AM9+AM4N/CsA	−0.03	0.82	−0.35	0.1
CsA C/D/kg (ng/ml/mg/kg)	AM1/CsA	−0.18	0.16	−0.41	0.049
	AM9/CsA	−0.12	0.34	−0.25	0.23
	AM4N/CsA	0.14	0.29	0.46	0.034
	DiH-CsA/CsA	0.04	0.75	0.04	0.87
	TriH-CsA/CsA	0.01	0.93	0.18	0.49
	dMC-CsA/CsA	−0.5	0.03	0.26	0.53
	AM1+AM9+AM4N/CsA	−0.18	0.16	−0.38	0.07
MPA C/D (ng/ml/g)	MPAG/MPA	−0.69	0.000001	−0.7	0.000001
	AcMPAG/MPA	−0.39	0.000001	−0.44	0.0009
	GluMPA/MPA	−0.43	0.000001	−0.41	0.001
	GluMPA+AcMPAG+MPAG/MPA	−0.69	0.000001	−0.7	0.000001
MPA C/D/kg (ng/ml/g/kg)	MPAG/MPA	−0.66	0.000001	−0.59	0.000001
	AcMPAG/MPA	−0.37	0.000001	−0.32	0.02
	GluMPA/MPA	−0.39	0.000001	−0.39	0.002
	GluMPA+AcMPAG+MPAG/MPA	−0.65	0.000001	−0.59	0.000001

C/D, dose-adjusted concentration; C/D/kg, dose/kg-adjusted concentration; Tac, tacrolimus; CsA, cyclosporine A; MPA, mycophenolic acid; ISD, immunosuppressive drug; AM1, 1-hydroxy cyclosporine; AM9, 9- hydroxy cyclosporine; AM4N, 4-N-demethyl cyclosporine; dMC-CsA, demethylcarboxylated cyclosporine metabolites; DiH-CsA, dihydroxylated cyclosporine metabolites; TriH-CsA, trihydroxylated cyclosporine metabolites; MI, 13-O-desmethyl tacrolimus; MIII, 15-O-desmethyl tacrolimus; MPA, mycophenolic acid; MPAG, phenyl glucuronide; AcMPAG, acyl glucuronide; GluMPA, glucoside

## Discussion

Observations revealing significant variation of immunoassays results evaluating concentrations of immunosuppressive drugs and variations of metabolites’ concentrations at the same parent drug concentrations are the reason for the need to seek new strategies of TDM. It has not yet been established with certainty whether and which ISD metabolites are relevant for the solid organ transplantation outcomes. There are some studies suggesting that they may be clinically significant [8,15,16]. Since the determination of concentrations of individual ISD metabolites or polymorphisms of genes encoding enzymes engaged in the metabolism of immunosuppressants are not widely available, C/D and C/D/kg evaluation seems to be a very promising strategy [6,7]. It is hypothesized that C/D and C/D/kg ratios reflect the immunosuppressive drug metabolism rate and hence the overall activity of enzymatic metabolic systems. With this assumption, one would expect that the higher dose-adjusted concentrations will be accompanied by lower production of individual metabolites and therefore lower MRs. However, we found that for tacrolimus and cyclosporine such relationships are not present in general. A completely different situation concerns MPA precursors. One possible explanation is the fact that the rate of formation of individual ISD metabolites and parent drug clearance depend not only on the activity of enzymes, but also on the activity of transport systems, mainly multidrug resistance 1 protein (MRP1) and multidrug resistance related protein (MRP) family. The different results in the case of pMPA may result from a significant impact on the analysed parameters of high concentrations of MPAG and different enzymatic, non-enzymatic and transport pathways involved in the pMPA pharmacokinetics.

It is suggested that faster metabolism in kidney transplant recipients receiving tacrolimus is associated with worse kidney graft function, worse prognosis and shorter graft survival [7]. Our research indicate that this is probably not directly associated with the presence of the major tacrolimus metabolites, i.e. MI and MIII. Similar conclusions can be drawn in regard to cyclosporine. Interestingly, in the case of pMPA, there is a direct link between C/D and C/D/kg ratios and concentrations of major MPA metabolites: GluMPA, AcMPAG and MPAG. Certainly, this requires further research considering the genetic variability of enzymes and transport proteins involved in the pharmacokinetics of immunosuppressants. It is suggested that MRs can be used as a surrogate of activity of cytochrome P450 isoenzymes indicating the presence of their specific polymorphisms [4,17]. Other authors indicate that a much more accessible parameters, such as C/D and C/D/kg ratios, can be used for this purpose [6]. In our study, we have showed that such strategy may probably be applied in the case of MPA precursors, but not in the case of cyclosporine nor tacrolimus. The research concerning the activity of enzyme and transport proteins involved in the pharmacokinetics of ISDs underlines the differences resulting from the presence of the transplanted organ: kidney or liver. The lack of relationship that was found in regard to CsA and Tac and the existence of correlations in the case of MPA were independent of the type of organ transplanted. The question of the influence of the ISD concentrations assessment methods on the obtained results also needs to be clarified, as many studies analysing dose-adjusted concentrations and pharmacogenetic studies used immunoassays [7,18].

In conclusion, easily available and easy to use in clinical practice C/D and C/D/kg ratios cannot be considered as parameters directly reflecting the rate of generation of major metabolites of cyclosporine and tacrolimus both in liver and kidney transplant recipients.

## Perspectives

Immunosuppressants’ dose-adjusted concentrations are proposed as measure of metabolism of parent drugs. This study was aimed at clarifying whether dose-adjusted and dose/kg-adjusted drug concentrations can be used as a measure of the generation of individual metabolites of tacrolimus, cyclosporine and mycophenolic acid.

- We have observed lack of correlation between dose-adjusted tacrolimus and cyclosporine concentrations and levels of metabolites; and high correlation for mycophenolic acid.
- Potential significance of the results.
- Tacrolimus and cyclosporine dose-adjusted concentrations cannot be used as surrogate of metabolites’ generation.

## Abbreviations

AcMPAG: acyl-MPA-glucuronide
C/D: dose-adjusted concentration
C/D/kg: dose/kg-adjusted concentration
CsA: cyclosporine A
DiH-CsA: dihydro-cyclosporine
dMC-CsA: desmethylcarboxy-cyclosporine
ESI: electrospray ionization mode
GluMPA: 7-MPA-glucoside
IQR: interquartile range
ISD: immunosuppressive drug
KTX: kidney transplantation
LC-MS/MS: liquid chromatography-tandem mass spectrometry
LTX: liver transplantation
MI: 13-O-desmethyl tacrolimus
MIII: 15-O-desmethyl tacrolimus
MPA: mycophenolic acid
MPAG: phenyl-MPA-glucuronide
MR: metabolic ratio
MRM: multiple-reaction monitoring
MUW: Medical University of Warsaw
pMPA: precursors of mycophenolic acid
SD: standard deviation
Tac: tacrolimus
TDM: therapeutic drug monitoring
TriH-CsA: trihydro-cyclosporine
